# Understanding the distinct role of PI3K(p110*α*) in cancer and the heart to prevent chemotherapy-induced cardiotoxicity: mechanisms and therapeutic opportunities

**DOI:** 10.3389/fcvm.2026.1891148

**Published:** 2026-08-24

**Authors:** Teleah G. Belkin, Yow Keat Tham, Amanda J. Edgley, Julie R. McMullen

**Affiliations:** 1 Baker Heart and Diabetes Institute, Melbourne, VIC, Australia; 2 School of Translational Medicine, Faculty of Medicine, Nursing and Health Sciences, Monash University, Melbourne, VIC, Australia; 3 Baker Department of Cardiovascular Research, Translation and Implementation, La Trobe University, Bundoora, VIC, Australia; 4 Baker Department of Cardiometabolic Health, The University of Melbourne, Melbourne, VIC, Australia; 5 Department of Medicine, The University of Melbourne, Melbourne, VIC, Australia; 6 Heart Research Institute, The University of Sydney, Newtown, NSW, Australia; 7 Department of Physiology, Monash University, Melbourne, VIC, Australia; 8 Monash Alfred Baker Centre for Cardiovascular Research, Faculty of Medicine, Nursing and Health Sciences, Monash University, Melbourne, VIC, Australia

**Keywords:** cancer, cardiotoxicity, doxorubicin, heart failure, PI3K

## Abstract

The development of new cancer therapies with greater efficacy has reduced mortality for many cancers, as well as improving survivorship. However, for some cancer treatments, this has uncovered unintended cardiotoxicity during or after treatment, and cardiovascular disease has emerged as a leading cause of mortality in some cancer survivors. This may occur because many of the same growth factor signaling pathways that lead to tumor growth are also critical for maintaining normal heart function. A class IA phosphoinositide 3-kinase, PI3K(p110*α*), is a critical regulator of normal heart biology, function and exercise-induced cardiac protection, but is also mutated/increased in some cancer types. In this review, we discuss the dual role of PI3K(p110*α*) signaling in cancer and the heart. The paradox being, that inhibiting PI3K(p110*α*) in a setting of some cancer types would be beneficial for halting/slowing tumor progression but could also damage heart muscle cells. Exercise and PI3K(p110*α*) has beneficial effects on heart function via cardiac excitation and contraction, mitochondrial adaptations, cellular stress responses to promote survival, preventing cell death and cardiac fibrosis. We contrast this to the chemotherapy, doxorubicin, which is crucial for the treatment of many cancers but negatively impacts many of the beneficial effects of exercise and PI3K(p110*α*). Doxorubicin's clinical efficacy is limited by dose-dependent cardiotoxicity including cardiomyopathy, atrial fibrillation, and heart failure. A summary of reported cardiovascular effects in clinical trials using PI3K inhibitors is also provided. Finally, we discuss some of the current and emerging approaches to mitigate cancer therapy-induced cardiotoxicity including pharmacological interventions, gene therapy and dietary approaches based on preclinical studies. Advances in the field are likely to come from continued refinement of cardiotoxicity models, the investigation of targeting molecular pathways and targets with relevant primary endpoints for both cancer and heart function, and collaborative research with discovery/preclinical scientists and clinicians.

## 1 Introduction

### 1.1 The emergence of cardiotoxicity during and after cancer treatment

Cancer is the second leading cause of death worldwide, contributing to approximately 10 million deaths in 2020 (1). With an increasing global prevalence of cancer, there is a concerted effort to develop novel therapies aimed at enhancing disease management. When identified in the early stages of progression, cancer is more likely to respond to treatment, leading to a greater probability of survival. Despite advancements in efficacy and prolonged survival rates from treatment, the use of cancer therapeutics remains a concern for both patients and clinicians due to potential side effects (2). A mounting concern has surfaced regarding the increasing incidence of cardiovascular disease (CVD) in cancer patients with oncological treatments such as chemotherapy and/or radiation. This concern stems from several factors including improved survival rates among cancer patients including children, the advancing age demographic, and the occurrence of pre-existing CVD conditions combined with genetic predispositions (3–5).

Cardiotoxicity can include any adverse effect of cancer therapies (e.g., chemotherapies or radiation) on cardiac structure and/or cardiac function. The heart damage can progress overtime, ultimately leading to conditions such as cardiomyopathy, heart failure (HF), and atrial fibrillation (AF) (6, 7). Cardiotoxicity can occur due to inflammation, oxidative stress, disruptions in signaling cascades, and direct damage to myocardial cells. The exact number of individuals affected by cardiotoxic complications resulting from cancer therapies remains unknown, however, estimates suggest that as many as 48% of patients have experienced these adverse effects (8–11).

### 1.2 Targeting cancer without damaging the heart

One of the challenges of protecting the heart while treating cancer is that many of the same growth factor signaling pathways that lead to cancer cell proliferation and growth are also critical for maintaining normal heart function and protecting it from pathology in settings of stress including hypertension, diabetes, and other cardiac insults (12). Here, we discuss in detail the regulation of a Class IA phosphoinositide 3-kinase [PI3K: PI3K(p110*α*)] because it is a critical regulator of normal heart size, cardiac function, and exercise-induced cardiac protection (12–17), as well as being mutated or increased in some cancer types (18–20). Furthermore, PI3K inhibitors have been developed to treat cancer, and other cancer drugs not specifically targeting PI3K can indirectly inhibit PI3K and processes it regulates.

The conundrum is that increased activation of the PI3K pathway is beneficial in the heart but can be detrimental in a setting of cancer where it should be inhibited to stop tumor progression. At the cellular and organelle level, exercise and PI3K(p110*α*) have been shown to have beneficial effects on heart function via cardiac excitation and contraction, mitochondrial adaptations, cellular stress responses to promote survival including the DNA damage response, preventing cell death and cardiac fibrosis. In this review, we contrast this to an anthracycline, doxorubicin (DOX), because anthracyclines are the most globally used and studied chemotherapies recognized to cause cardiotoxicity (21) and negatively impact many of the same beneficial adaptations of exercise and PI3K(p110*α*). We provide a summary of reported cardiovascular effects in clinical trials using PI3K inhibitors and discuss new therapeutic approaches to mitigate cancer therapy-induced cardiotoxicity.

## 2 Overview of PI3Ks

PI3Ks are a diverse group of lipid enzymes that phosphorylate the 3′-OH group of phosphatidylinositols (PtdIns) on the plasma membrane leading to activation of cellular signaling pathways involved in physiological processes including cellular proliferation and metabolic homeostasis (19). However, the dysregulation of the PI3K signaling pathway has been associated with a range of pathologies including inflammation, CVD, diabetes, immunodeficiency, and cancer (19, 20).

Based upon their structural characteristics, regulation, and lipid substrate specificity, PI3Ks have been categorized into three major classes (Class I, II, III) (22, 23). The most extensively characterized class of PI3Ks in relation to cancer and the heart is Class IA PI3Ks. Class IA PI3Ks comprise of a catalytic subunit (p110*α*, p110*β*, and p110*δ*) and regulatory subunit (p85). In response to a stimulus (e.g., nutrients, growth factors) the association of the catalytic and regulatory subunit with tyrosine kinase receptors and tyrosine-phosphorylated adaptor proteins initiates the activation of PI3K (24, 25). PI3K(p110*α*) is the main focus of this review.

### 2.1 PI3K(p110*α*) and cancer

**Figure 1:**
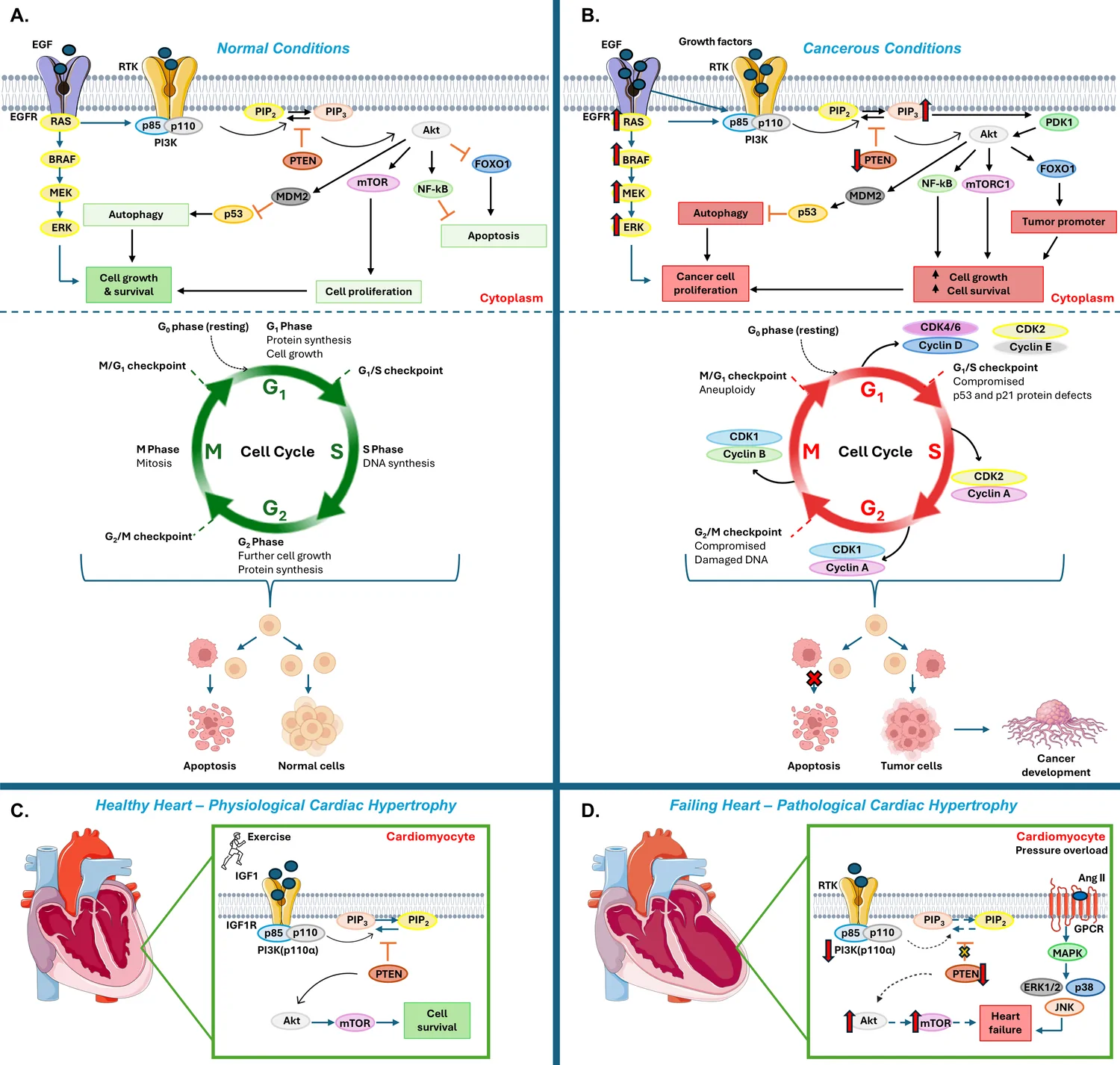
PI3K signaling in a setting of cancer and cardiac hypertrophy **(A)** Under normal conditions, extracellular signals activate RTKs, leading to RAS activation. Activated RAS signals through BRAF to phosphorylate MEK, which in turn activates ERK. This facilitates healthy cell division and maturation. In normal conditions, RTKs recruit p85 (regulatory subunit) and p110 (catalytic subunit) to form PI3K. Activated PI3K converts PIP2 into PIP3, which serves as a second messenger to recruit and activate Akt. Akt coordinates downstream signaling to promote cellular homeostasis including cell cycle progression, cell growth and survival. FOXO1 activity is inhibited leading to apoptosis and cell cycle control, mTOR promotes controlled cell proliferation. Akt modulates MDM2 activity which regulates p53 turnover, maintaining normal cell cycle checkpoints. The cell cycle is divided into two major phases: interphase and M phase. Interphase is the phase during which the cell prepares for division and is further subdivided into three phases: G1, S, and G2. M phase involves mitosis where the nucleus divides and chromosomes are replicated and then distributed into two nuclei. Cells undergo apoptosis if a mutation is detected. **(B)** Increased activation of the ERK/ MAPK pathway via receptor tyrosine kinases e.g. EGF binding to EGFR is a key feature of many cancers (121). Overactivation of the MAPK pathway, often driven by oncogenic mutations, results in uncontrolled cell proliferation and tumor progression (122). Activation of RTKs in cancer cells recruits p85 and p110, converting PIP2 into PIP3. PIP3 recruits and activates PDK1, which in turn phosphorylates and activates Akt. Akt promotes oncogenic signaling via multiple downstream targets. Loss of PTEN further increases PIP3 accumulation, sustaining Akt signaling and driving uncontrolled proliferation, survival, and tumor progression. These combined effects disrupt normal interphase checkpoints (G1, S, G2) and M phase, resulting in accelerated cell cycle progression and tumorigenesis. **(C)** In response to stimuli such as exercise, IGF1 binds to IGF1R on the cardiomyocyte plasma membrane, triggering the recruitment of p85 and p110. The interaction of p110 and p85 is critical for PI3K(p110*α*) activity. Upon activation, p85 inhibition is removed, allowing p110 to phosphorylate PIP2 and generate PIP3. This lipid molecule then functions as a second messenger, activating both Akt-dependent and Akt-independent signaling pathways critical for cell survival and growth. **(D)** Persistent activation of PI3K drives excessive production of PIP3, resulting in sustained Akt-dependent and Akt-independent signaling. This maladaptive signaling promotes pathological cardiomyocyte growth, metabolic dysfunction, and increased cell death. The loss of PTEN further amplifies these processes, contributing to the development of pathological cardiac hypertrophy (123). Dotted lines indicate the indirect pathway through which reduced PI3K(p110*α*) activity may contribute to pathological cardiac remodeling. Pressure overload and release of factors such as Ang II leads to activation of GPCR and activation of downstream MAPK pathways (JNK, p38 MAPK, ERK1/2). These signaling pathways promote cardiomyocyte hypertrophy, fibrosis, and inflammation, ultimately leading to contractile dysfunction and heart failure. Akt, Protein kinase B; Ang II, Angiotensin II; BRAF, V- Raf murine sarcoma viral oncogene homolog B; EGF, Epidermal growth factor; EGFR, EGF receptor; ERK, Extracellular signal-regulated kinases; FOXO1, Forkhead box protein O1; GPCR, G protein-coupled receptor; mTOR, mammalian target of rapamycin; MAPK; Mitogen-activated protein kinase pathway; MDM2, Mouse double minute 2 homolog; MEK, Mitogen-activated protein kinase; NF-kB, Nuclear factor kappa-light-chain-enhancer of B cells; PDK1, Phosphoinositide-dependent protein kinase 1; PTEN, Phosphatase and tensin homolog; RTK, Receptor tyrosine kinase.

Abnormal activation of the PI3K pathway drives tumorigenesis by promoting excessive cell proliferation, migration, and survival. PI3K(p110*α*) encoded by PIK3CA and its antagonist phosphate and tensin homolog (PTEN) are frequently mutated in a setting of cancer (18, 26, 27). Under basal conditions, PIP3 is temporarily induced following growth factor stimulation and subsequently degraded by lipid phosphatases, including PTEN, which dephosphorylates PIP3 to PIP2 to terminate PI3K signaling. However, in cancer cells, increased levels of PIP3 result from increased activity of oncogenic signaling proteins upstream of PI3K or PI3K mutations, leading to sustained signaling (19) (Figure 1). Missense mutations can occur in all domains of p110*α* (28, 29), but they predominantly cluster into two “hotspots”: E542K and E545K mutations within the helical domain, and H1047R within the kinase domain (30, 31). These mutations lead to persistent activation of p110*α*, as confirmed by cell culture and murine models demonstrating their roles in tumor initiation and maintenance (32–36). Helical domain mutations reduce the inhibition of p110*α* by p85 or facilitate direct interactions of p110*α*, while kinase domain mutations enhance its interactions with lipid membranes (37). Subsequently, there is a concerted effort to develop inhibitors targeting Class IA PI3K, primarily to treat cancers driven by p110*α* mutations or by the downstream activation of Class IA PI3K resulting from mutations or amplification of RTKs (38, 39). However, because PI3K plays a critical role in cardiac physiology, such inhibitors may induce cardiotoxic effects (as described below).

### 2.2 PI3K(p110*α*) and the heart

Among the Class IA PI3K subgroup, the p110*α* isoform has emerged as a pivotal regulator influencing heart size, including exercise-induced cardiac enlargement (physiological cardiac hypertrophy) and protection (15, 17, 40). The first study to show that PI3K was a critical regulator of organ size was in a genus of flies (Drosophila). The upregulation of Dp110, the Drosophila PI3K homolog, induced the enlargement of ocular and wing structures. Conversely, reduced expression of the Dp110 variant resulted in smaller structures (41). The involvement of PI3K activity specifically in the regulation of cardiac size stemmed from experiments involving transgenic mice expressing a cardiac-specific constitutively active (ca) form of PI3K(p110*α*) to increase PI3K(p110*α*) activity or a dominant negative (dn) form of PI3K(p110*α*) to reduce PI3K(p110*α*) activity. The model is cardiomyocyte specific due to transgene expression being driven by the *α*-myosin heavy chain promoter (15, 42). In the hearts of heterozygous caPI3K transgenic mice, a 6.5-fold increase in PI3K activity was observed, correlating with a 20% increase in heart size compared to non-transgenic (Ntg) control mice. This phenotypic expression resembled heart enlargement referred to as physiological cardiac hypertrophy. Physiological cardiac hypertrophy is characterized by an increase in heart size with normal or enhanced heart function. This differs from pathological cardiac hypertrophy, which is associated with depressed heart function, excessive collagen deposition (cardiac fibrosis), inadequate angiogenesis and typically progresses to HF (43, 44). In contrast to physiological cardiac hypertrophy in caPI3K, the hearts of heterozygous dnPI3K transgenic mice with decreased PI3K activity (77%), had smaller hearts compared to Ntg mice (17% reduction) (42). Under basal conditions, both caPI3K and dnPI3K transgenic mice demonstrated normal cardiac function, and dnPI3K mice displayed blunted cardiac enlargement/hypertrophy to exercise training for four weeks (15). Exercise training in mice has been shown to increase PI3K(p110*α*) activity in the heart (45) and both exercise and increased PI3K activity have been shown to exhibit cardioprotective effects. Swim training from four weeks of age in a mouse model with dilated cardiomyopathy (DCM) increased lifespan in both male and female mice by approximately 20% and 16%, respectively (14). To explore the influence of altered PI3K activity on lifespan, this DCM model was genetically crossed with caPI3K (increased PI3K activity) or dnPI3K (decreased PI3K activity) mouse models. In a sedentary setting, DCM-caPI3K mice experienced a longer lifespan compared to the DCM model. In contrast, the DCM-dnPI3K model showed a significant reduction in lifespan (14). Another study investigated exercise-induced cardiac protection in a cardiac disease model of pressure overload induced by ascending aortic banding. Prior to aortic banding, mice were subjected to four weeks of swim training (17). The exercise training attenuated cardiac dysfunction and the development of cardiac fibrosis as well as attenuated pathological cardiac hypertrophy in Ntg mice. In contrast, no protection was identified in exercise trained dnPI3K mice that underwent aortic banding, indicating that PI3K plays a critical role for exercise-induced protection (17). A large body of evidence has shown that caPI3K mice are protected in settings of cardiac stress whereas dnPI3K mice are more susceptible. The dnPI3K mouse model demonstrated a consistent display of accelerated HF and pathological responses to pressure overload, myocardial infarction, AF, and diabetic cardiomyopathy. In contrast, the caPI3K mouse model was protected against cardiac dysfunction and showed reduced pathology in response to the aforementioned cardiac stress models (14, 17, 46, 47). PI3K(p110*α*) signaling appears to play a critical role in governing heart rhythm and function by modulating cardiac ion channels (48–51). Collectively, these studies emphasize the importance of PI3K in promoting normal cardiac growth, its involvement in exercise-induced heart growth and protection, and its crucial protective role in various cardiac stress settings.

### 2.3 Evidence of cardiotoxicity in cancer patients receiving PI3K inhibitors

Based on the critical role of PI3K(p110*α*) in the heart described above, there is the potential for PI3K inhibitors to cause cardiotoxicity in cancer patients. PI3K inhibitors are classified into three main categories based on their isoform selectivity: Pan-PI3K inhibitors, isoform-selective PI3K inhibitors, and dual PI3K/mTOR inhibitors. Pan-PI3K inhibitors target all four class I isoforms (Class IA: p110*α*, p110*β*, p110*δ* and Class IB: p110*γ*). However, their lack of isoform specificity often results in varying degrees of toxicity and off-target effects. In contrast, isoform-selective PI3K inhibitors offer greater specificity towards individual PI3K isoforms. This selectivity allows for a more favorable therapeutic window and improved tolerability, particularly in malignancies driven by specific PI3K isoforms. Dual PI3K/mTOR inhibitors are designed to carry out therapeutic effects on both PI3K and mTOR pathways by targeting all p110 isoforms along with the mTOR catalytic domain. Given the high degree of sequence homology between the mTOR catalytic domain and the p110 catalytic subunit, some PI3K inhibitors also demonstrate mTOR inhibitory activity. These dual inhibitors can effectively overcome feedback activation commonly observed with mTORC1 inhibitors when used alone (52, 53).

A summary of recent clinical trials of PI3K inhibitors is provided in Table 1. Although cardiotoxicity was not a primary concern with earlier PI3K inhibitors such as Copanlisib and Duvelisib, cardiovascular events including AF and hypertension have been reported, though these were generally infrequent and not considered dose-limiting. However, caution should be taken. The extent of cardiovascular risk is likely to be influenced by dosage, treatment duration, and the patient's cardiovascular health. Preclinical studies highlighted that mice with reduced cardiac PI3K(p110*α*) displayed greater cardiotoxicity under settings of cardiac stress (described in Sec. 2.2). Thus, cancer patients with underlying cardiovascular disease or risk may be at greater risk of PI3K inhibitor-induced cardiotoxicity. Of interest, Novartis recently acquired a pan-mutant-selective PI3K(p110*α*) inhibitor (SNV4818) from Synnovation Therapeutics for $2 billion to advance breast cancer treatment. Current PI3K inhibitors such as Piqray and Itovebi target both mutant and endogenous forms of PI3K(p110*α*), contributing to adverse effects including hyperglycemia (54, 55). In contrast, the novel drug SNV4818 is designed to selectively inhibit mutant PI3K(p110*α*), potentially improving safety and tolerability, and is being explored in early clinical trials in combination with existing therapies (56).

**Table 1:** Overview of recent clinical trials on PI3K inhibitors.

PI3K inhibitor name	Study title	Cancer type	Phase	Reported cardiovascular adverse effects			Class
Samotolisib^#^ Started: 28/11/2017 Completed: 1/12/2024 Number of patients: 18 (1 death) Eligibility Age: 12 months to 21 years	*Samotolisib in Treating Patients with Relapsed or Refractory Advanced Solid Tumors, Non-Hodgkin Lymphoma, or Histiocytic Disorders with TSC or PI*3*K/MTOR Mutations (A Pediatric MATCH Treatment Trial)* (110)	Solid tumors, non-Hodgkin lymphoma, or histiocytic disorders	II	HF—1/17 (5.88%) Sinus bradycardia—1/17 (5.88%) Sinus tachycardia—2/17 (11.76%) Hypertension—1/17 (5.88%) Hypotension—1/17 (5.88%)			Dual PI3K/mTOR Class I PI3K
Buparlisib^#^ Started: 29/09/2014 Completed: 1/01/2022 Number of patients: 23 Eligibility Age: 18+ years	*Phase Ib Study of BKM*120 *With Cisplatin and XRT in High Risk Locally Advanced Squamous Cell Cancer of Head and Neck* (111)	Locally advanced squamous cell carcinoma of the head and neck	I	40 mg BKM120 administered daily for 45 days. Cisplatin: Dose 30 mg/m^2^, given IV, weekly on days: (1, 8, 15, 22, 29, 36 and 43)	40 mg BKM120 administered daily for 45 days. Cisplatin: Dose 35 mg/m^2^, given IV, weekly on days: (1, 8, 15, 22, 29, 36 and 43)		Pan Class I PI3K
				AF—1/17 (5.88%) Palpitations—1/17 (5.88%) Sinus bradycardia—2/17 (11.76%) Hypertension—5/17 (29.41%)	Sinus bradycardia—1/16 (16.67%)		
Izorlisib^#^ Started: 20/07/2020 Completed: 27/02/2024 Number of patients: 29 Eligibility Age: 18+ years	*MEN*1611 *With Cetuximab in Metastatic Colorectal Cancer (CPRECISE-* 01*)* (112)	Metastatic Colorectal Cancer	I + II	Phase 1b: Dose Confirmation	Phase II: Cohort Expansion		PI3K(p110*α*)-Isoform Class I PI3K
				Sinus tachycardia—1/7 (14.29%) Supraventricular tachycardia −1/7 (14.29%)	Cardio-respiratory arrest—1/22 (4.55%) Hypotension—1/22 (4.55%)		
Umbralisib^#^ Started: 01/10/2016 Completed: 01/07/2019 Number of patients: 13 Eligibility Age: 18+ years	*TGR-*1202 *and Ibrutinib in Treating Patients With Relapsed or Refractory Diffuse Large B-Cell Lymphoma* (113)	Diffuse large B-cell lymphoma	II	AF—1/13 (7.69%) Sinus tachycardia—1/13 (7.69%)			PI3K(p110*δ*)-Isoform Class I PI3K
Alpelisib^#^ Started: 21/05/2014 Completed: 20/05/2017 Number of patients: 17 Eligibility Age: 18+ years	*BYL*719* + T-DM*1 *in HER*2*(+) Metastatic Breast Cancer Pts Who Progress on Prior Trastuzumab & Taxane Tx* (114)	Metastatic breast cancer	I	Cohort 1: (250 mg Alpelisib, 3.6 mg/kg T-DM1)	Cohort 2: (300 mg Alpelisib, 3.6 mg/kg T-DM1)		PI3K(p110α)-Isoform Class I PI3K
				Hypertension—1/11 (9.09%) LV systolic dysfunction—1/11 (9.09%) Sinus bradycardia—1/11 (9.09%) Sinus tachycardia—5/11 (45.45%) Hypertension—1/11 (9.09%) Palpitations—1/11 (9.09%)	Hypertension—1/6 (16.67%) Sinus tachycardia—2/6 (33.33%) Hypertension—2/6 (33.33%)		
Duvelisib Started: 17/06/2013 Completed: 18/11/2020 Number of patients: 129 Eligibility Age: 18+ years	*Study of Duvelisib in Participants With Refractory Indolent Non-Hodgkin Lymphoma* (115)	Indolent Non-Hodgkin lymphoma, marginal zone lymphoma, small lymphocytic lymphoma	II	AF—2/129 (1.55%) Cardiac failure—1/129 (0.78%) Cardiac failure congestive—1/129 (0.78%) Coronary artery occlusion—1/129 (0.78%) Pericarditis—1/129 (0.78%) Cardio-respiratory arrest—1/129 (0.78%) Hypertension—7/129 (5.43%) Hypotension—7/129 (5.43%)			Dual PI3Kδ, *γ* Class I PI3K
Duvelisib Started: 01/12/2013 Completed: 12/06/2020 Number of patients: 90 Eligibility Age: 18+ years	*Phase* 3 *Extension Study of Duvelisib and Ofatumumab in Participants With CLL/SLL Previously Enrolled in Study IPI-*145*-*07 (116)	Chronic lymphocytic leukemia, Small lymphocytic lymphoma	III	Acute myocardial infarction—1/90 (1.11%) Cardiac failure—1/90 (1.11%) Cardiac failure chronic—1/90 (1.11%) Atrioventricular block—1/90 (1.11%) Aortic dissection—1/90 (1.11%) Arterial rupture—1/90 (1.11%) Vena cava thrombosis—1/90 (1.11%) Hypertension—2/90 (2.22%)			Dual PI3Kδ, γ Class I PI3K
Copanlisib Started: 19/11/2012 Completed: 18/05/2023 Number of patients: 226 Eligibility Age: 18+ years	*Open-label, Uncontrolled Phase II Trial of Intravenous PI*3*K Inhibitor BAY*80*-*6946 *in Patients With Relapsed, Indolent or Aggressive Non-Hodgkin's Lymphomas* (117)	Indolent or aggressive Non-Hodgkin's lymphoma (NHL), chronic lymphocytic leukemia (CLL)	II	Part A: Indolent NHL/CLL	Part A: Aggressive NHL	Part B: Indolent NHL	Pan Class I PI3K
				Hypertension—1/33 (3.03%)	Acute myocardial infarction—1/51 (1.96%) Circulatory collapse—1/51 (1.96%) Aortic valve incompetence—1/51 (1.96%)	AF—2/142 (1.41%)	
Parsaclisib Started: 14/03/2018 Completed: 07/06/2024 Number of patients: 126 Eligibility Age: 18+ years	*A Study of INCB*050465 *in Relapsed or Refractory Follicular Lymphoma* (118)	Relapsed or refractory follicular lymphoma	II	Treatment A: Parsaclisib 20 mg daily for 8 weeks followed by 20 mg weekly up to 1 year	Treatment B: Parsaclisib 20 mg daily for 8 weeks followed by 2.5 mg weekly up to 1 year		PI3K(p110δ)-Isoform Class I PI3K
				Hypotension—1/23 (4.35%)	AF—1/103 (0.97%) Pericardial effusion—1/103 (0.97%) Sinus bradycardia—1/103 (0.97%) Supraventricular tachycardia—1/103 (0.97%) Hypertension—1/103 (0.97%)		

# denotes clinical trials with <30 patients; caution should be taken with interpretation of % of cardiovascular events due to low numbers.

Adverse events were graded by the National Cancer Institute's Common Terminology Criteria for Adverse Events version 4.0. Dual PI3K inhibitor: targets both the PI3K and mTOR enzymes. Pan PI3K inhibitor: inhibits all four catalytic isoforms of class I PI3K (Class IA: p110α, p110*β*, p110δ, and Class IB: p110γ). Isoform: specifically targets individual PI3K isoforms rather than the entire PI3K family.

## 3 Anthracycline-induced cardiotoxicity: stages of toxicity and mechanisms of action

Anthracyclines are a group of chemotherapeutics well known to have cardiotoxic properties. They are recognized for their efficacy but restricted in the clinic by dose-dependent adverse effects. Anthracyclines are implicated in inducing irreversible myocardial damage, with reports of ∼5% of cancer survivors treated with anthracyclines subsequently developing chronic HF (57–60). Anthracycline-induced cardiotoxicity (AIC) has been divided into three classes (Table 2). Of these, acute cardiotoxicity and early-onset chronic progression are the most commonly encountered (61–65). Pathologically, features of acute AIC include inflammatory infiltration, cardiomyocyte injury, and interstitial oedema (61, 66). Chronic AIC is characterized histopathologically by fibrosis, myofibrillar dropout, vacuolation, and necrosis (21, 67).

**Table 2:** Anthracycline-induced cardiotoxicity categories.

Category	Onset	Characteristics	Course
Acute cardiotoxicity	Occurs after a single dose; within 14 days/after Anthracycline treatment	Decreased myocardial contractility ECG alterations and arrhythmias	Reversible
Early onset chronic progression	Within 1 year of Anthracycline treatment completion	Dilated cardiomyopathy LV dysfunction Reduced LVEF HF (approximately 5% of patients #)	Irreversible
Late onset chronic progression	>1 year after Anthracycline treatment completion	Dilated cardiomyopathy Cytoplasmic vacuolization of cardiomyocytes Mitochondrial dysfunction Cardiomyocyte death Decrease in LVEF (approximately 7–20% of patients #) HF (Up to 48% of patients #)	Irreversible

LVEF, Left ventricular ejection fraction. Information in the table comes from the following references (11, 59, 84, 119, 120). # - dependent on dose and risk factors.

### 3.1 Mechanisms via which AIC induces cardiotoxicity

Topoisomerase II (Top2) is a principal target of AIC in cancer cells. Top2 is a nuclear enzyme crucial for managing DNA tangles and supercoils by cleaving both strands of the DNA helix during replication and transcription. Anthracyclines intercalated into DNA form a stable anthracycline-DNA-Top2 ternary complex, thereby inhibiting the enzyme's activity. This ternary complex prevents the resealing of breaks in double stranded DNA. Consequently, anthracyclines cause irreversible DNA damage, leading to genomic instability and eventual apoptotic cell death in rapidly dividing cancer cells. However, given Top2 is present in cardiomyocytes, its inhibition by anthracyclines can also damage cardiomyocyte DNA and have long term effects on the heart, resulting in cardiomyopathy (68). Studies have also shown that anthracyclines, in particular, DOX, form an irreversible association with cardiolipins, a specific lipid class present only within the inner mitochondrial membrane (69, 70). This can ultimately lead to mitochondrial and cardiac dysfunction (58, 71).

**Figure 2:**
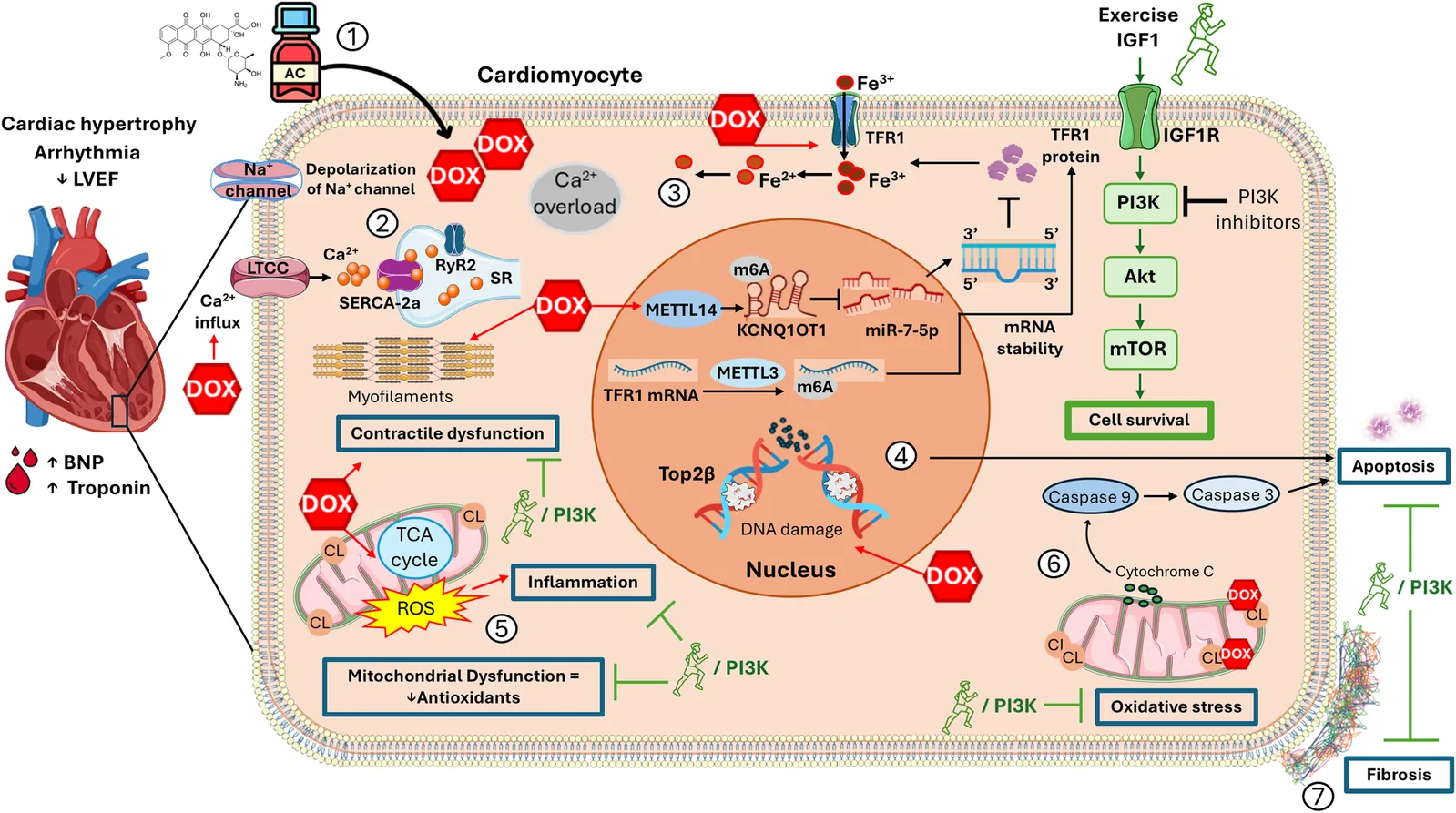
The opposing actions of DOX (damage, red) and PI3K signaling (protection, green) on critical cellular and molecular mechanisms in the heart. Mechanisms of DOX: (1) DOX enters the cardiomyocyte primarily through passive diffusion, accumulating in the cytoplasm and mitochondria. (2) Following depolarization via sodium channels, calcium enters the cardiomyocyte through L-Type calcium channels (LTCC). This influx of calcium activates ryanodine receptors (RyR2) on the SR, a specialized receptor that detects intracellular calcium and initiates further calcium release from the SR. DOX impairs calcium reuptake into the SR by altering the function of SERCA2a and may also destabilize RyR2, leading to calcium leak. The resulting calcium overload contributes to contractile dysfunction and cardiomyocyte injury. (3) DOX causes iron accumulation by enhancing TFR1-mediated iron uptake. It upregulates METTL3 and METTL14 expression, which facilitates m6A methylation and increases the stability of TFR1 mRNA and KCNQ1OT1. KCNQ1OT1 downregulates miR-7–5p, thereby further upregulating TFR1 expression, contributing to excessive iron accumulation. These regulatory mechanisms have also been involved in oncological and neurological contexts, where they are implicated in tumor progression and dysregulated iron homeostasis (124). (4) DOX accelerates DNA mutations by inhibiting Top2*β*. This activates the DNA damage response and triggers apoptosis. (5) Oxidative stress damages mitochondrial DNA and reduces ATP production, further amplifying ROS generation. (6) Mitochondrial dysfunction triggers the release of cytochrome c, activating caspase-dependent apoptotic pathways, leading to cell death. (7) Chronic inflammation and loss of cardiomyocytes contribute to fibrosis, structural remodeling, and impaired cardiac function, ultimately leading to heart failure. Exercise stimulates IGF1 secretion, activating IGF1R and recruits PI3K. PI3K phosphorylates PIP2 to PIP3 at the plasma membrane, promoting recruitment and activation of Akt. This pathway protects the heart via multiple mechanisms (highlighted in green). AC, anthracycline; BNP, B-type natriuretic peptide; Ca^2+^, calcium; CL, cardiolipin; DNA, deoxyribose nucleic acid; DOX, doxorubicin; IGF1, insulin-like growth factor 1; Fe^2+^, ferrous iron; Fe^3+^, ferric iron; LTCC, L-type calcium channel activation; LVEF, left ventricular ejection fraction; PI3K, phosphoinositide 3-kinase; mTOR, mammalian target of rapamycin; ROS, reactive oxygen species; RyR2, ryanodine receptor 2; SR, sarcoplasmic reticulum; TFR1, transferrin receptor protein 1.

DOX is now recognized to cause damage to cardiac myocytes via multiple mechanisms (Figure 2). It enters cardiomyocytes primarily via passive diffusion, accumulating within the cytoplasm and mitochondria where it undergoes redox cycling, disrupting the electron transport chain and producing excessive reactive oxygen species (ROS). Following depolarization through sodium channels, calcium enters the cell via L-type calcium channels (LTCC), activating ryanodine receptors (RyR2) on the sarcoplasmic reticulum (SR) to trigger further calcium release. DOX impairs calcium reuptake into the SR by altering SERCA2a function and may destabilize RyR2, causing calcium leaks that lead to calcium overload, contractile dysfunction, and cell injury (72). Additionally, DOX promotes iron accumulation by enhancing TFR1-mediated iron uptake, upregulating METTL3 and METTL14 expressions to facilitate m6A methylation, which stabilizes TFR1 mRNA and KCNQ1OT1, further elevating TFR1 levels and contributing to iron overload (73). DOX accelerates DNA mutations by inhibiting Top2*β*, activating the DNA damage response and inducing apoptosis. The resulting oxidative stress damages mitochondrial DNA, reduces ATP production, and amplifies ROS generation, while mitochondrial dysfunction leads to cytochrome c release and activation of caspase-dependent apoptotic pathways, resulting in cell death. Chronic inflammation and cardiomyocyte loss drive fibrosis, structural remodeling, and impaired cardiac function, ultimately resulting in HF (Figure 2).

### 3.2 The role of PI3K in regulating DOX-induced cardiotoxicity

**Figure 3:**
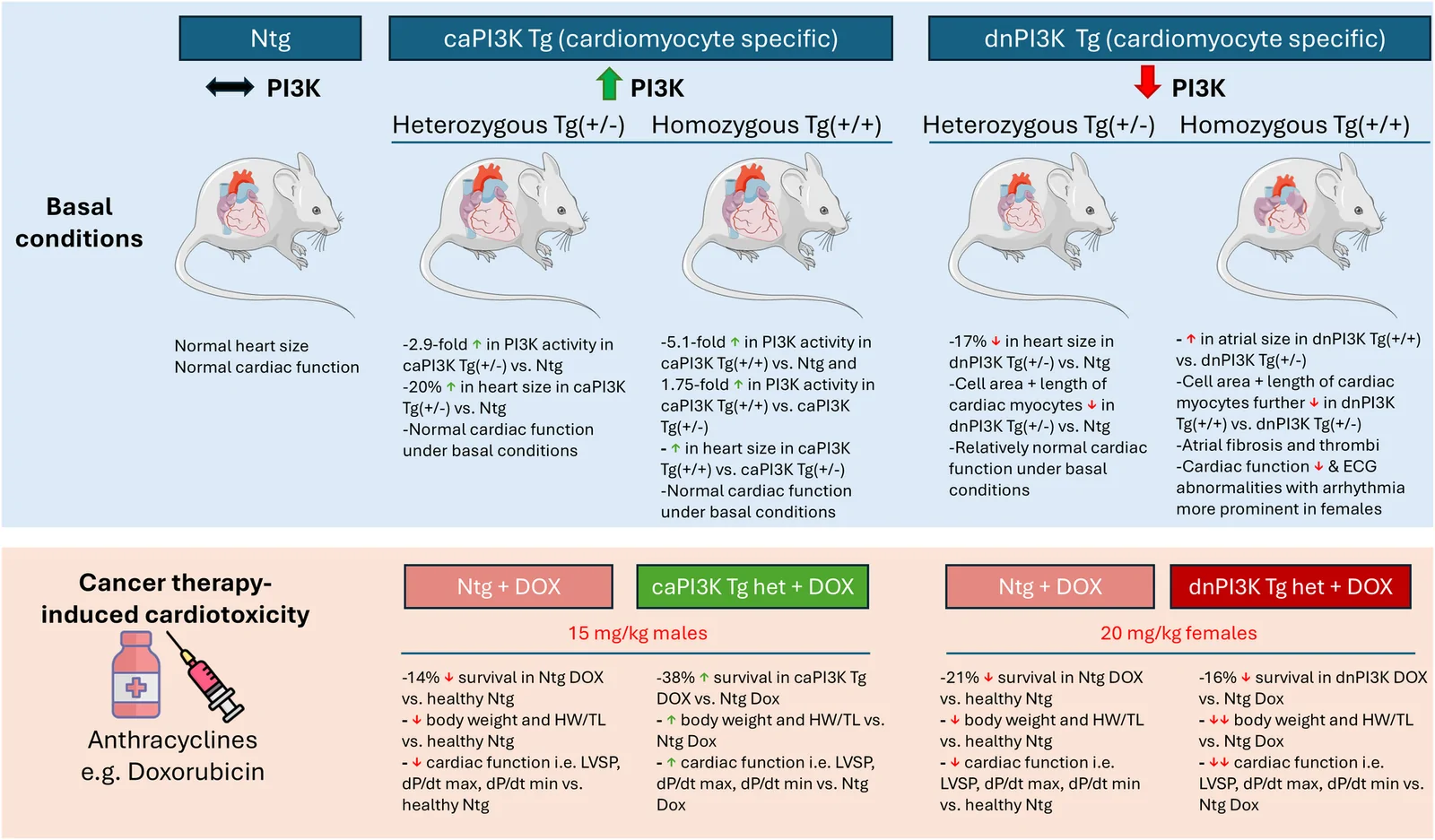
PI3K is a critical regulator of cardiac growth and function and provides protection against DOX. Under basal conditions, Ntg mice exhibit normal heart size and function. Cardiac-specific expression of a constitutively active PI3K transgene (caPI3K) increases PI3K activity and induces heart enlargement resembling physiological cardiac hypertrophy. In homozygous caPI3K, there is a 5.1-fold increase in PI3K activity vs. Ntg and 1.75-fold increase and further increase in heart size vs. caPI3K heterozygous (13). Conversely, expression of a dominant-negative PI3K transgene (dnPI3K), associated with a 77% reduction in PI3K activity, results in smaller hearts relative to Ntg (42). Neither model exhibits cardiac dysfunction or pathology. Homozygous dnPI3K mice exhibit increased atrial size compared to heterozygous dnPI3K. Atrial fibrosis and thrombus formation are evident, accompanied by reduced cardiac function and ECG abnormalities. Arrythmias are more pronounced in females (13). Under conditions of cancer therapy-induced cardiotoxicity, caPI3K male mice administered a single dose of DOX (15 mg/kg) show increases in survival, body weight, heart weight/tibia length (HW/TL), and improvements in cardiac function when compared to Ntg DOX mice. dnPI3K female mice administered a single dose of DOX (20 mg/kg) show decreases in survival, body weight, HW/TL, and significant decline in cardiac function when compared to Ntg DOX mice (74). caPI3K, constitutively activated PI3K; DOX, doxorubicin; dnPI3K, dominant negative PI3K; HW/TL, heart weight/tibia length, LVEF, left ventricular ejection fraction; LVSP, left ventricular systolic pressure; Ntg, non-transgenic.

The multiple mechanisms via which exercise and the PI3K pathway mediate cardiac protection in contrast to the cardiotoxicity with DOX are highlighted in Figure 2. The role of PI3K in regulating DOX-induced cardiotoxicity has been directly assessed by studying DOX-treated cardiac myocyte specific transgenic mice with increased PI3K (caPI3K) and decreased PI3K (dnPI3K). In caPI3K male mice administered a single dose of DOX (15 mg/kg), cardiac function was improved vs. Ntg DOX mice with no mortality observed two weeks after DOX administration, suggesting that the expression of the caPI3K transgene is beneficial in a setting of cardiotoxic stress. By contrast, in female dnPI3K mice administered a single dose of DOX (20 mg/kg), cardiac dysfunction and mortality was declined vs. Ntg DOX. These findings indicate that reduced PI3K exacerbates DOX-induced cardiac dysfunction (74) (Figure 3).

### 3.3 Identifying cancer patients at greatest risk of cardiotoxicity with PI3K inhibitors and anthracyclines

Guidelines for identifying and stratifying patients at greatest risk of cancer-therapy induced cardiotoxicity via imaging (e.g., echocardiography, cardiac magnetic resonance) and circulating biomarkers (e.g., cardiac troponin, natriuretic peptides) have been described in detail previously (limited value found with biochemical biomarkers in particular) (4, 7, 75). Here, we have focused on potential new approaches related to PI3K. The PI3K pathway which lies downstream of IGF1 and insulin is known to be defective/dysregulated in settings of obesity, diabetes, age and underlying CVD in mice and humans (51, 76–78). We previously identified lipid species related to the IGF1-PI3K pathway which were decreased or dysregulated in cardiac tissue or plasma of patients with diabetes and/or CVD or those who developed cardiac complications later (13, 77). We propose such markers may identify patients at risk of CVD complications including AF, HF and stroke (79). Thus, PI3K inhibitors, anthracyclines, and other cancer drugs [e.g., BTK inhibitors (80)] which inhibit protective physiological mechanisms may put those with underlying PI3K defects due to obesity, diabetes and CVD at even greater risk. Whether circulating lipids related to the PI3K pathway could identify cancer patients at elevated risk is yet to be formally assessed. Mining an open-access cardiac profiling data set from PI3K transgenic mice shed some light on cardiac toxicity of another cancer drug targeting clusterin (clusterin gene expression regulated by PI3K), and may be a useful approach to assess whether inhibiting new cancer drug targets is likely to lead to cardiac toxicity (81).

## 4 Therapeutic approaches for preventing or treating cancer therapy-induced cardiotoxicity

Methods to prevent and treat cardiotoxicity involve approaches reducing the cardiotoxic impact of cancer drugs directly, and pharmacological interventions aimed at reducing cardiotoxicity more generally (4, 7, 66, 75, 82–84). A list of cardioprotective agents used in the clinic during cancer therapy or after cardiotoxicity has been described in detail previously within guidelines/recommendations (5, 7). However, common CVD medications (e.g., *β*-blockers, AT_1_ receptor blockers, and statins) have not consistently shown a clear benefit for cancer therapy-induced cardiotoxicity (7, 85, 86).

Standard CVD medications are typically introduced after DOX-induced damage has occurred but are not consistently reliable preventive therapies. Dexrazoxane (DEX) is a clinically approved cardioprotective drug for DOX-induced cardiotoxicity (7, 87). However, its earlier use remains controversial due to concerns that it may compromise DOX's anti-tumor efficacy and increase the risk of secondary malignancies (88). Consequently, there is no broadly accepted preventive therapy that protects the heart while remaining compatible with effective chemotherapy.

### 4.1 New approaches targeting cancer-therapy induced cardiotoxicity based on animal studies

**Figure 4:**
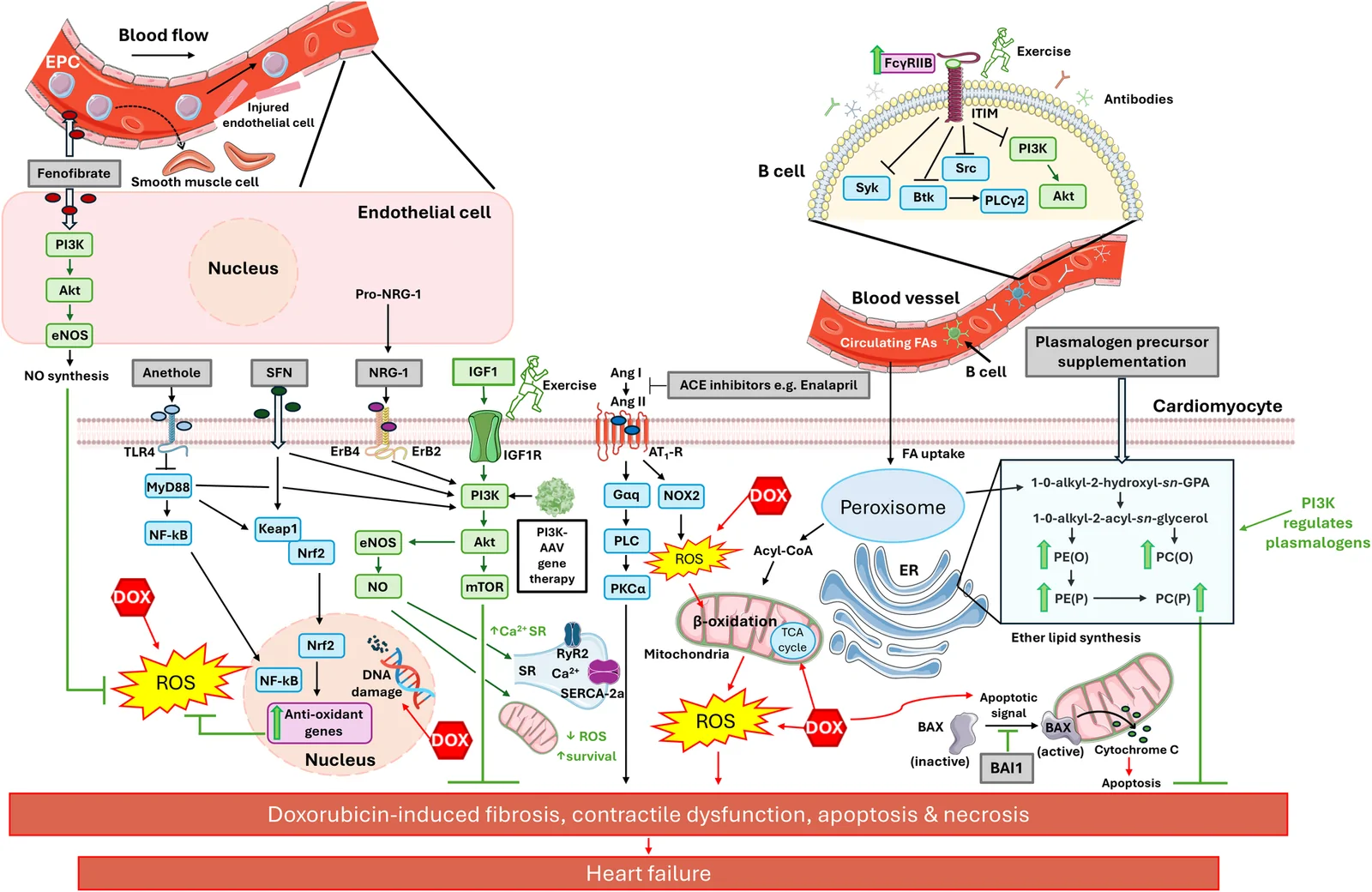
Overview of current drugs and targeted signaling pathways in endothelial cells and cardiomyocytes for the attenuation of DOX-induced cardiotoxicity. Approaches directly related to PI3K signaling are highlighted in green. Other drugs/approaches are shown in grey. Fenofibrate upregulates endothelial nitric oxide synthase (eNOS) and further enhances nitric oxide production in the endothelial cell. Fenofibrate also increases the circulation of EPCs, which may enable the repair of injured vascular cells (125). Anethole suppresses the TLR4/MyD88/NF-kB pathway and enhances the Nrf2 and PI3K pathways. This in turn reduces inflammation, ROS, and apoptosis (91). Sulforaphane (SFN) enters the cardiomyocyte and interacts with Keap1. This prevents the degradation of Nrf2 and results in triggering nuclear translocation by Nrf2 phosphorylation and increasing antioxidant genes (90). NRG-1 regulates cardiomyocyte structure, viability, and proliferation. After binding to ErB4/ErB2, different signaling pathways are activated including the PI3K pathway and the phosphorylation of eNOS, which leads to increased NO production (74). Exercise induces IGF1 secretion, which activates the IGF1 receptor leading to the recruitment of PI3K. The activation of IGF1/PI3K/Akt pathway mediates cardioprotective effects such as the inhibition of pathological cardiac remodeling (12). AAV PI3K gene therapy increases PI3K activity in cardiomyocytes (126). Enalapril, an ACE inhibitor, prevents the formation of Ang II and suppresses ROS production, preserving mitochondrial function (92). Exercise increases FC*γ*RIIB expression which regulates B cell signaling transduction and function, raising the activation threshold of B cells. Upregulated B cells enable an anti-inflammatory role, resulting in protection against DOX-induced cardiotoxicity (93). Plasmalogen precursor supplementation bypasses the rate limiting step of plasmalogen biosynthesis within the peroxisome, enabling increased production of ether lipids known as alkyl or alkenyl-phosphatidylethanolamine [PE(O) and PE(P)] and alkyl or alkenyl-phosphatidylcholine [PC(O) and PC(P)]. Plasmalogen precursor supplementation is able to attenuate pathological features such as cardiac remodeling (109). BAI1 (small molecule inhibitor of BAX) inhibits BAX activation and conformational changes required for its translocation from the cytosol to the mitochondria, therefore, preventing the release of cytochrome c and the initiation of apoptosis (96). AAV, adeno-associated virus; ACE, angiotensin II converting enzyme; Akt, protein kinase B; Ang I/II, angiotensin I/II; AT_1_R, angiotensin II type 1 receptor; BAI1, BAX activation inhibitor 1; Btk, Bruton's tyrosine kinase; DOX, doxorubicin; eNOS, endothelial nitric oxide synthase; EPC, endothelial progenitor cells; ER, endoplasmic reticulum; ErB2/4, erythroblastic leukemia viral oncogene homolog 2/4; FA, fatty acid; G*α*q, G protein alpha; IGF, insulin-like growth factor; IGF1R, insulin-like growth factor 1 receptor; ITIM, immunoreceptor tyrosine-based inhibition motif; Keap1, Kelch-like ECH associating protein 1; mTOR, mammalian target of rapamycin; MyD88, myeloid differentiation protein 88; NF-kB, nuclear factor-kB; NO, Nitric oxide; NOX2, nicotinamide adenine dinucleotide phosphate oxidase 2; Nrf2, nuclear factor erythroid 2-realted factor 2; NRG-1; neuregulin-1; PI3K, Phosphoinositide 3-kinase; PKA, PKC, protein kinase C; ROS, reactive oxygen species; SFN, sulforaphane; Src, proto-oncogene tyrosine-protein kinase; Syk, spleen tyrosine kinase; TLR4, toll-like receptor 4.

DOX-induced cardiotoxicity has been the most widely studied in animal models to date and is the focus of this section. A summary of rodent studies investigating new approaches with potential cardioprotective properties in the context of DOX-induced cardiotoxicity is provided in Table 3 and Figure 4, as well as current approaches in the clinic (e.g., ACE inhibitors and exercise). Approaches which have shown some benefit based on cardiac function and/or life span in rodent studies include Neuregulin 1, Fenofibrate, Sulforaphane, Anethole, a small molecule inhibitor of BAX, ACE inhibitors (e.g., Enalapril) and exercise (74, 89–96). At least two of the exploratory agents have entered clinical trials (fenofibrate: completed with benefits reported; sulforaphane: recruitment phase) (97, 98), ACE inhibitors and exercise have been assessed in randomized clinical trials. For ACE inhibitors, evidence of benefit is limited (7, 99) while for exercise there is reasonable evidence suggesting cardiac protection (7, 100). Other approaches yet to be formally tested but worthy of exploration include those with the capacity to positively impact signaling pathways regulated by exercise such as PI3K. The rationale for this lies in the evidence that both exercise and increased PI3K(p110*α*) have been implicated in providing benefit in a setting of DOX-induced cardiotoxicity (74, 101, 102). Cardiac-selective PI3K(p110*α*) gene therapy has been shown to provide protection in cardiac disease mouse models such as pressure overload and diabetic cardiomyopathy (17, 103, 104), but has yet to be tested in a model of DOX-induced cardiotoxicity. Another approach worthy of investigation may be the supplementation of plasmalogen precursors. A study reported in an abstract, showed that plasmalogen supplementation provided protection against DOX-induced cognitive impairment in C57Bl/6J mice (105). Chemotherapy-induced cognitive impairment (“chemobrain”) due to inflammation and oxidative stress in the brain is recognized clinically (106, 107). In relation to the heart, we previously showed there was a reduction in plasmalogen levels in dnPI3K and DCM transgenic mouse hearts with reduced PI3K activity (108). Supplementation with key plasmalogen precursors batyl, selachyl, and chimyl alcohols restored major cardiac plasmalogen species (p18:0, p18:1, and p16:0), and improved cardiac, lung, and renal outcomes in DCM mice, with greater benefit in males. These benefits were associated with reduced ceramides, increased tetralinoleoyl cardiolipins, and enhanced mitochondrial electron transport chain supercomplex proteins, suggesting improved mitochondrial health (109). Given that oxidative stress, mitochondrial function, and lipid dysregulation also occur in DOX-induced cardiotoxicity, restoring plasmalogen levels may represent a promising strategy to protect against cardiac injury (Figure 4).

**Table 3:** Studies assessing cardioprotective agents or exercise in rodents administered doxorubicin to induce cardiotoxicity.

Study	Animal model	Sex	Treatment & intervention when applicable	Summary
*Neuregulin* 1 *Improved Cardiac Function in Doxorubicin-Treated Mice with Cardiomyocyte-Specific Overexpression of a Dominant-Negative PI*3*K p*110*α* (74)	Mouse FVBN/J background Cardiac-myocyte specific caPI3K Tg (↑PI3K in cardiac myocytes) Cardiac-myocyte specific dnPI3K Tg (↓PI3K in cardiac myocytes) caPI3K WT (male) dnPI3K WT (female) *n* = 8–13/group	Male (caPI3K) & Female (dnPI3K) 12 weeks of age	DOX: Male: Single dose (15 mg/kg) Female: Single dose (20 mg/kg) Neuregulin 1 (NRG1): Given as a concurrent treatment with DOX (0.75 mg/kg) in female dnPI3K mice	Survival: Two-week survival was significantly reduced to 14% in WT-DOX male mice vs. WT (100%) and caPI3K (100%) male mice. caPI3K expression improved survival to 38% in caPI3K-DOX-treated mice vs. WT-DOX (14%) male mice. DOX reduced two-week survival to 21% in WT-DOX and 16% in dnPI3K-DOX female mice. NRG1 improved survival to 44% in NRG1-WT-DOX female mice and 48% in NRG1-dnPI3K-DOX female mice. Cardiac function: WT-DOX male mice had lower LVSP, dP/dt max and dP/dt min than WT, and this was prevented in caPI3K-DOX. Female dnPI3K-DOX mice had lower HR, LVSP, dP/dt max and dP/dt min than WT-DOX and NRG1 largely prevented this. Conclusion & Mechanism: The results showed that PI3K(p110α) is important for protecting the heart against DOX. One of the primary signaling cascades activated by NRG1-HER interaction is the PI3K pathway. NRG1-HER signaling plays a crucial role in protecting the heart against various forms of cardiac stress. Activation of PI3K is essential for several effects of NRG1 (74). NRG1 helps with the partial loss of PI3K(p110α) to improve cardiac function in DOX-injured hearts. The NRG1 gene belongs to the EGF family.
*Fenofibrate*^#^ *Attenuates Doxorubicin-Induced Cardiac Dysfunction in Mice* via *Activating the eNOS/EPC Pathway* (89)	Mouse^^^ FVBN/J background *n* = 4–13/group	Male 8 weeks of age	DOX: Weekly dose (4 mg/kg) for 5 weeks Fenofibrate: Oral feeding gavage for 4 weeks (100 mg/kg/d) + L-NAME (40 mg/kg/d)	Survival: No deaths observed. Cardiac function: EF and FS were significantly decreased in the DOX group (EF: 85%, FS: 45%) vs. Saline control group (EF: 95%, FS: 58%). EF and FS were significantly decreased in DOX + Fenofibrate + L-NAME group (EF: 85%, FS: 48%) vs. DOX + Fenofibrate group (EF: 90%, FS: 52%). Conclusion & Mechanism: Treatment with fenofibrate led to cardioprotection against DOX. This was associated with an attenuated inflammatory response and improved LV function (89). Fenofibrate activates Akt/eNOS and vascular endothelial growth factor signaling, leading to the stimulation of endothelial progenitor cell pathways and mitigation of DOX-induced cardiac toxicity (89).
Exercise* *Inhibits Doxorubicin-Induced Cardiotoxicity* via *Regulating B Cells* (93)	Mouse C57Bl/6J background *μ*MT^−/−^ (lacks B cells. Received exercise-derived splenic B cells) FcγRIIB^−/−^ (lacks the inhibitory Fc gamma receptor IIB protein) *n* = 6–8/group	Male 8 weeks of age	DOX: Weekly dose (5 mg/kg) for 4 weeks Exercise: treadmill running—60 min/day at 15 m/min for 8 weeks (6 weeks prior were duration increases and acclimatization)	Survival: Not reported. Cardiac function: 7 days after the last dose of saline or DOX, EF and FS were significantly reduced in DOX-treated mice (EF: 43%, FS: 20%) vs. saline-treated control mice (EF: 65%, FS: 38%). EF and FS were significantly improved in the DOX-treated + ExT (EF: 57%, FS: 25%) vs. DOX-treated control mice (EF: 43%, FS: 20%). Conclusion & Mechanism: Highlights the protective effects of exercise through the regulation of B cell response in cardiotoxicity (93). Exercise showed protective effects on B cells by the adoptive transfer of splenic B cells from exercised donor mice to μMT-/- mice. Enhanced FcγRIIB regulates B cell signaling transduction and function by raising the activation threshold of B cells. These threshold-upregulated B cells play an anti-inflammatory role, being protective against DOX-induced cardiotoxicity.
*Exercise Training Preserves Myocardial Strain and Improves Exercise Tolerance in Doxorubicin-Induced Cardiotoxicity* (94)	Mouse C57Bl/6J background *n* = 7–20/group	Male 8 weeks of age	DOX: Weekly dose (5 mg/kg) for 5 weeks Exercise: treadmill running—40 min per session, 4 days/week for 5 weeks	Survival: Exercise training did not affect survival in DOX-treated mice. Cardiac function: LVEF was reduced by 26% in DOX-treated mice and 22% in mice treated with DOX + Exercise training vs. control. Conclusion & Mechanism: Suggests that a low-to-moderate intensity aerobic exercise training program performed concurrently with DOX treatment does not prevent a reduction in LVEF but mitigates cardiac atrophy, preserves myocardial strain, and improves exercise tolerance (94).
*Exercise Intervention Decreases Acute and Late Doxorubicin-Induced Cardiotoxicity* (95)	Mouse C57Bl/6J background Balb/c (immunocompetent) Nude (T-cell deficient) *n* = 8–10/group	Female 4 weeks of age	DOX: Twice a week (2.5 mg/kg) for 2 weeks Exercise: treadmill walking—45 min/day, 5 days/week, 12 m/min	Survival: 67% for control mice, 22% for DOX-treated mice, and 56% for DOX-treated + Exercise training mice at 100 days post myocardial infarction. Cardiac function: 90 days after DOX administration, EF and FS were significantly reduced in DOX-treated mice (EF: 42%, FS: 23%) vs. DOX + Exercise training mice (EF: 57%, FS: 32%). 45 days after MI surgery, EF and FS were significantly reduced in DOX-treated mice (EF: 23%, FS: 14%) vs. DOX + Exercise training mice (EF: 40%, FS: 22%). Control mice showed no differences in EF and FS at either timepoints (EF: 60%, 40% and FS: 33%, 23%). Conclusion & Mechanism: The mechanism(s) responsible for reduced cardiotoxicity in exercised mice was not the main focus of the study. However, it is suggested that cardioprotective effects of exercise may be linked to its ability to reduce ROS generation in the heart (95).
*Sulforaphane (SFN)*^#^ *Protects the Heart from Doxorubicin-Induced Toxicity* (90)	Mouse^^^ 129/sv (used to examine the cardiac effects of SFN administration during chronic DOX treatment) *n* = 4–12/group	Male 14–16 weeks of age	DOX: Weekly dose (5 mg/kg) for 4 weeks Sulforaphane (organic compound found in vegetables): Weekly dose (4 mg/kg) for 5 days/week for 5 weeks	Survival: 8% for DOX-treated mice, 75% for SFN + DOX mice, and 100% for SFN and control mice. Cardiac function: DOX caused a significant fall in heart function vs. baseline pre-DOX (EF: 78% to 68%, FS: 46% to 37%) and this was prevented in the group with SFN: SFN + DOX (EF: 78% to 74%, FS: 45% to 43%). Conclusion & Mechanism: SFN treatment preserved cardiac function and significantly reduced DOX-induced cardiomyopathy and mortality in mice (90). SFN is a potent Nrf2-activating agent and has been recognized as a chemopreventive.
*Anethole Alleviates Doxorubicin-Induced Cardiac and Renal Toxicities: Insights from Network Pharmacology and Animal Studies* (91)	Rat Wistar Albino *n* = 6/group	Male 4–6 weeks of age	DOX: Once daily (2.5 mg/kg) for 2 weeks Anethole (organic compound found naturally in plants): Once daily (orally—125 mg/kg) for 2 weeks Once daily (orally—250 mg/kg) for 2 weeks	Survival: 65% for DOX-treated rats, 85% for DOX + Anethole (125 mg/kg) rats, and 100% for DOX + Anethole (250 mg/kg) and control rats. Cardiac function: In DOX-treated rats, ST elevation, QT interval, and R-R interval were significantly higher and QRS complex, P wave, and HR were significantly lower in DOX-treated rats vs. control rats. Rats treated with DOX + Anethole (125 mg/kg or 150 mg/kg) had significantly higher QRS complex, P wave, and HR and significantly lower ST elevation, QT interval, and R-R interval vs. DOX-treated rats. Conclusion & Mechanism: Anethole can counteract DOX-induced oxidative stress, inflammation, lipid peroxidation, and apoptosis, highlighting its potential as a preventive strategy against DOX-induced cardiotoxicity (91). Anethole alleviated oxidative stress by increasing antioxidant enzyme activity and decreasing inflammatory cytokines.
Pretreatment with Angiotensin-Converting Enzyme* *Inhibitor Improves Doxorubicin-Induced Cardiomyopathy* via *Preservation of Mitochondrial Function* (92)	Rat Sprague Dawley *n* = 8/group	Female 10 weeks of age	DOX: Weekly dose (cumulative dose of 25 mg/kg) for 6 weeks Enalapril (ACE inhibitor): Administered in drinking water (10 mg/kg/d for each animal). Started 1 week before the first DOX injection and continued for an additional 3 weeks after the final DOX injection	Survival: 75% for DOX-treated rats and 100% for the DOX + Enalapril and control rats after 10 weeks. Cardiac function: FS was significantly decreased in DOX-treated rats (25%) but attenuated with Enalapril (31%). Control rats had a FS of 40%. Conclusion & Mechanism: Enalapril administration mitigates DOX-induced cardiac dysfunction. Enalapril administration preserves mitochondrial respiratory efficiency and reduces DOX-associated free radical production (92).
*A Small-Molecule Allosteric Inhibitor of BAX Protects Against Doxorubicin-Induced Cardiomyopathy* (96)	Mice BAX knockout (KO) WT *n* = 4–15/group	Male (8–15 weeks of age) & Female (8–15 weeks of age)	DOX: Long term dose: (3 mg/kg) for 2 weeks (cumulative dose of 24 mg/kg) to BAX KO (C57Bl/6J) male & female mice Single dose: 20 mg/kg to C57Bl/6N male & female mice BAI1: Long term dose: 2 mg/kg to male & female mice	Survival: Long term dose—Male: WT-saline (13%), WT-DOX (60%), BAX KO saline (0%), BAX KO DOX (50%), Female: WT-saline (0%), WT-DOX (0%), BAX KO saline (0%), BAX KO DOX (25%). Male: saline (13%), DOX (50%), DOX + BAI1 (40%), Female: saline (0%), BAI1 (0%), DOX (13%), DOX + BAI1 (0%). Single dose—Male: saline (0%), DOX (15%), DOX + BAI1 (4%). Female: saline (0%), BAI1 (0%), DOX (0%), DOX + BAI1 (7%). Cardiac function: Long term dose—FS was significantly decreased in WT-DOX-treated mice (28%) vs. WT-saline mice (36%). No differences in FS in DOX-treated BAX KO (33%) vs. saline BAX KO mice (33%). FS was significantly improved in DOX-treated BAX KO (33%) vs. WT-DOX treated mice (28%). Long term dose + BAI1 (2 mg/kg)—FS was significantly decreased in DOX-treated mice (27%) vs. saline mice (34%). No differences in FS were observed between DOX + BAI1 and saline. FS was significantly improved in DOX + BAI1 mice (34%) vs. DOX-treated mice (27%). Single dose DOX + BAI1 (2 mg/kg)—At Day 5, in females, FS was significantly reduced in DOX-treated mice (24%) vs. saline (31%). Mice treated with DOX + BAI1 showed improvement (31%) vs. DOX (24%). In males, FS was significantly decreased in DOX-treated mice (24%) vs. saline (31%). Mice treated with DOX + BAI1 showed a significant improvement (30%) vs. DOX-treated mice (24%). Conclusion & Mechanism: BAI1 inhibits BAX-dependent apoptosis and necrosis, therefore, preventing DOX-induced cardiomyopathy (96).

# Denotes agents/strategies which have entered clinical trials, *denotes strategies assessed in randomized trials, ^^^ signifies studies in which an animal group(s) are <6 and results may be underpowered.

caPI3K, Constitutively active PI3K; dnPI3K, Dominant-negative PI3K; DOX, Doxorubicin; EF, Ejection fraction; FS, Fractional shortening; HR, Heart rate; LV, Left ventricle; LVEF, Left ventricular ejection fraction; LVEDP, Left ventricle end diastolic pressure; LVSP, Left ventricle systolic pressure; NRG1, Neuregulin 1, SFN, Sulforaphane; WT, Wild-type.

## 5 Conclusions and perspectives

The emergence of new therapies for cancer has reshaped treatment options, and in many cases increased patient survival. Concurrently, the identification of cancer therapy-induced cardiotoxicity in some patients has posed challenges; in severe cases, necessitating the cessation of cancer treatment regimens. Preclinical and clinical studies have shed some light on the mechanisms of cardiotoxicity, but further work is required. There remains an urgent need for cardioprotective strategies and personalized medicine approaches to mitigate cardiotoxicity without compromising the efficacy of essential cancer treatments.

For any cancer treatment targeting a critical pathway/regulator for heart biology and function, it is essential to consider the potential impact of the cancer drug on adversely impacting the cardiovascular system. Here, we provide the example of PI3K(p110*α*) which has been targeted in the cancer and cardiovascular fields. The conundrum is that patients can often present with both cancer and underlying CVD. In some cancer types the goal would be to inhibit PI3K signaling, but in CVD, the goal would be to increase PI3K. By contrast, reducing PI3K is likely to make the heart more susceptible to cardiac disease and failure. Continued research is therefore essential to better understand the context-dependent effects of PI3K modulation, identify predictive biomarkers, and guide the development of therapies that maximize anti-tumor actions while minimizing cardiovascular adverse outcomes.

Animal studies have provided some insight and uncovered some strategies for further investigation (such as Neuregulin 1, Fenofibrate, Sulforaphane, Anethole, BAX inhibitor and selectively targeting PI3K in the heart). While these approaches have shown some promise, it is important to acknowledge the limitations of animal studies. The extrapolation of these results to humans must be approached with caution due to species-specific differences in metabolism, immune responses, and cardiac physiology. There are also no standard experimental models for cancer therapy-induced cardiotoxicity. As an example, there has been variability in DOX treatment protocols (administration and dose e.g., i.p. vs. i.v., single vs. multiple and cumulative dose) and assessment of cardiotoxicity. Often therapies and approaches have been tested in animal models with chemotherapies but in the absence of cancer itself. It is essential to examine whether a cardioprotective agent impacts tumorigenesis and the efficacy of the chemotherapy being assessed. Additionally, while rodent models are effective for studying acute interventions and short-term cardiotoxicity, they may not fully capture long-term outcomes or the chronic effects of low-dose chemotherapy exposure common in clinical settings. Nonetheless, these preclinical studies form a foundation for translational research. With refinements in cancer models incorporating treatment-induced cardiotoxicity it is hoped that preclinical studies will assist in informing future clinical trials to prevent heart damage during and after cancer treatment.
